# Enhancing visual brain-computer interface through V1-targeted RTMS by modulating visual attention

**DOI:** 10.1162/IMAG.a.1013

**Published:** 2025-11-17

**Authors:** Xinyi Zhang, Shengpei Wang, Ying Gao, Yijun Wang, Shuang Qiu, Huiguang He

**Affiliations:** Laboratory of Brain Atlas and Brain-Inspired Intelligence, State Key Laboratory of Brain Cognition and Brain-Inspired Intelligence Technology, Institute of Automation, Chinese Academy of Sciences, Beijing, China; Intelligent Computing Infrastructure Lab, Lenovo Research, Beijing, China; School of Future Technology, University of Chinese Academy of Sciences (UCAS), Beijing, China; State Key Laboratory on Integrated Optoelectronics, Institute of Semiconductors, Chinese Academy of Sciences, Beijing, China

**Keywords:** BCI, EEG, SSVEP, rTMS, visual cortex

## Abstract

Brain-computer interfaces (BCIs) enable users to control devices directly through brain activity. Despite recent advancements in machine-learning algorithms, the signal-to-noise ratio (SNR) of the brain’s responses still limits decoding performance, highlighting the necessity for targeted neuromodulation techniques to overcome this limitation. To evaluate whether 5 Hz repetitive transcranial magnetic stimulation (rTMS) targeting the primary visual cortex (V1) can enhance SSVEP-based BCI performance by improving neural signal SNR and modulating visual network dynamics. Twenty-four healthy subjects underwent both real and sham rTMS in a randomized order. The rTMS was precisely implemented through magnetic resonance imaging (MRI)-guided navigation to stimulate V1 in participants. Electroencephalograms (EEGs) were recorded during SSVEP tasks and resting-state before, immediately after, and 20 min after rTMS. SSVEP tasks were conducted across four frequency bands: low frequency (LF: 8–12 Hz), middle frequency (MF: 18–22 Hz), high frequency (HF: 28–32 Hz), and super high frequency (SHF: 38–42 Hz). The discriminability of BCI commands in the MF (+7.53%) and HF (+11.4%) bands significantly improved (p<0.001 ), driven by enhanced prominence of both fundamental and harmonic components (p<0.01 ). Quantitative analysis indicated that the improved SNR was due to the suppression of the background activity (p<0.05 ). This effect was linked to rTMS-induced enhancements in visual attention, evidenced by increased occurrence and contribution of microstate B during the SSVEP task (p<0.01 ). This study highlights the potential of 5 Hz rTMS as an effective neuromodulatory tool for optimizing BCI performance, particularly through facilitating visual attention.

## 1 Introduction

Visual evoked potentials (VEPs) have garnered considerable interest in brain-computer interface (BCI) research, which establishes a direct communication pathway between the brain and external devices. Among these, steady-state VEPs (SSVEPs) are stable neural responses elicited by periodic visual stimuli. SSVEPs exhibit consistent frequency components that correspond to the visual input, along with harmonic components arising from flickering lights at varying frequencies (Gao et al., 2014). Such characteristics enable SSVEPs at distinct frequencies to effectively encode users’ explicit focus on specific spatial locations. Therefore, SSVEPs provide significant advantages for constructing control systems (D. Zhang et al., 2022; S. Zhang et al., 2022) and have proven valuable in clinical neurosciences research (Chota et al., 2024; Nie et al., 2023) and disease diagnosis (Krishnan et al., 2005; Zheng et al., 2021).

Studies have demonstrated that visual stimuli within the 1–90 Hz range can evoke SSVEP responses with varying intensities (Gu et al., 2024; Herrmann, 2001). Researchers have developed SSVEP systems with specific encoding frequency bands tailored for diverse applications (X. Chen, Wang, Nakanishi, et al., 2015; Jiang et al., 2022; Ming et al., 2023; Xu et al., 2020). Stimuli in the 8–16 Hz range are frequently employed in BCIs due to their relatively strong responses, which yield a high signal-to-noise ratio (SNR). Such high SNR enhances the discriminability of SSVEP targets (Gu et al., 2024), thereby improving BCI decoding performance. Conversely, higher flicker frequencies tend to evoke weaker responses with lower SNR, rendering target differentiation more challenging (Gu et al., 2024). Nonetheless, these frequencies are preferred for constructing user-friendly BCIs (X. Chen et al., 2023; Ming et al., 2021) due to their reduced flicker perception (Gu et al., 2024). To achieve fast and accurate target recognition, researchers have refined spatial filters to enhance signal similarity between SSVEP and target templates (X. Chen, Wang, Gao, et al., 2015; B. Liu et al., 2021; Nakanishi et al., 2017), or applied deep learning techniques to extract task-relevant components for specific pattern recognition (J. Chen et al., 2023; Waytowich et al., 2018; X. Zhang et al., 2024). While such frameworks have improved the distinct features of SSVEP for decoding, the SNR of the EEG signal ultimately limits decoding performance (Shi et al., 2024), as this cannot be addressed solely through algorithmic advancements. Therefore, improving SNR at the signal level is imperative, necessitating alternative approaches to optimize system performance.

Neuromodulation induces various behavioral effects, including enhanced motor skill learning, altered cognitive control and decision-making, memory, language, and visuospatial abilities (Grover et al., 2022; Kosnoff et al., 2024; Vatrano et al., 2023). However, its application in BCI research remains in the early stages. Transcranial electrical stimulation (TES) and transcranial magnetic stimulation (TMS) are established non-invasive neuromodulation techniques with demonstrated efficacy in modulating visual functions. Compared to TES, TMS offers distinct advantages in focality and provides immediate phenomenological indicators of successful stimulation, such as contralateral motor movements (for motor cortex stimulation) or phosphene perception (for visual cortex stimulation). The minimum TMS intensity that can induce phosphene perception is called the phosphene threshold (PT), and lower PT indicates higher cortical excitability (Antal et al., 2003; Brighina et al., 2002; Gothe et al., 2002). These attributes make TMS particularly valuable for investigating neurophysiological effects (Lefaucheur et al., 2014). High-frequency repetitive TMS (rTMS) has been associated with subjective improvements in visual functions, including visual field recovery in stroke patients (El Nahas et al., 2019) and enhanced contrast sensitivity in individuals with amblyopia (Thompson et al., 2008). In healthy individuals, rTMS has been shown to improve target visibility (Romei et al., 2010) and visual feature discrimination (Waterston & Pack, 2010). On the other hand, SSVEPs reflect the brain’s objective response to external stimuli. Although rTMS has demonstrated subjective benefits for visual function, its neuronal mechanisms remain unclear, as does its potential to facilitate the translation of visual-evoked brain activity into actionable commands in BCIs. Therefore, investigating the effects of rTMS on SSVEPs holds significant scientific potential.

This study examines whether rTMS targeting the primary visual cortex (V1) can enhance BCI performance by modulating neural activity prior to an SSVEP-based task. The SSVEP task included four frequency bands: low frequency (LF: 8–12 Hz), middle frequency (MF: 18–22 Hz), high frequency (HF: 28–32 Hz), and super high frequency (SHF: 38–42 Hz). EEG analyses were conducted during the SSVEP task and resting state to identify potential neural mechanisms underlying possible performance improvements in the SSVEP task. We hypothesized that rTMS enhances SSVEP-BCI decoding performance, and that this improvement is mediated by increased SNR of the EEG signals.

Our results demonstrate that V1-targeted rTMS significantly improved decoding performance in the MF and HF bands compared to the sham condition, alongside a significantly enhanced task-related component prominence in their corresponding bands. Although SSVEP components in the SHF band were also enhanced, this did not translate into improved decoding performance. The observed enhancement in task prominence was attributed to the suppression of task-unrelated components rather than an increase in task-related components. The increase in microstate B in the task state following rTMS suggests that rTMS facilitated visual attention during target selection. Furthermore, this increase in microstate B during SSVEP tasks was negatively correlated with both the phosphene threshold and resting-state power. These findings suggest that stronger modulation of the visual processing network is associated with heightened cortical excitation, and the changes induced by rTMS are interrelated between the task and resting states. This study highlights V1-targeted rTMS as an effective approach to improving SSVEP-based BCI performance by suppressing the task-unrelated components with enhanced visual attention.

## 2 Methods

### 2.1 Participants

Twenty-four healthy volunteers were recruited (mean age: 24.88 ± 1.56 (SD) years; range: 20–27; M/F: 13/11). None reported a history of mental or neurological diseases or a family history of the above conditions. All participants had normal or corrected-to-normal vision. Our study complies with all relevant ethical regulations regarding human research. The experiment was approved by the institutional review board of the Institute of Automation, Chinese Academy of Sciences (NO. IA21-2310-020302). Participants were compensated at a rate of 100 Yuan per hour.

### 2.2 Experimental design

**Fig. 1.**
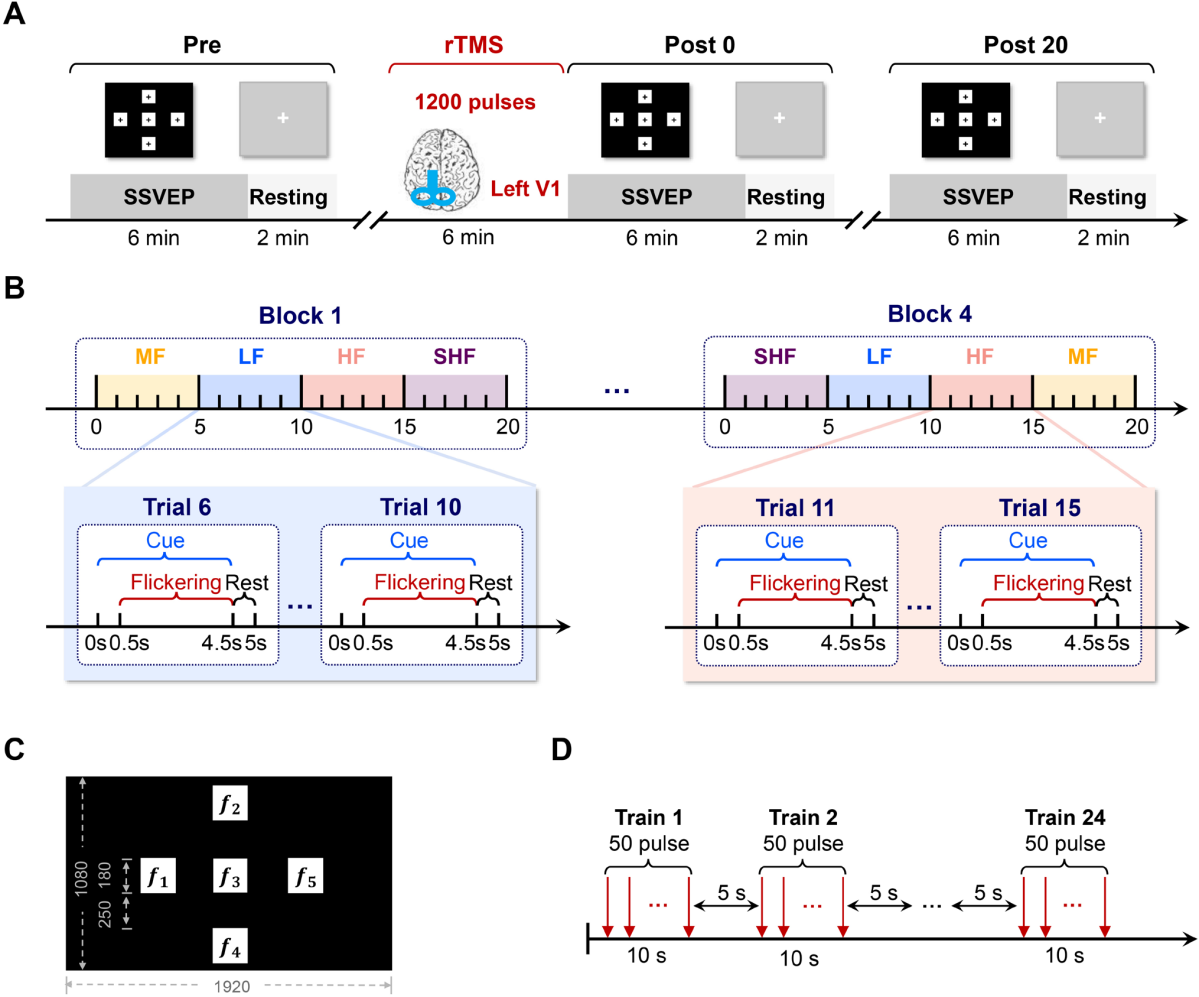
Experimental design of the study. Each participant completed two sessions, separated by a minimum interval of 1 week. The procedures for both sessions were identical, except for the rTMS condition, which was pseudo randomly assigned as either real or sham stimulation. (A) Session procedure: SSVEPs and resting-state EEG were recorded at three time points: before rTMS (Pre), immediately after rTMS (Post0), and 20 min after rTMS (Post20). (B) SSVEP task: The SSVEP task consisted of four blocks, each separated by a 15-s break. Within each block, four frequency bands were presented in randomized order, with five targets cued randomly within each frequency band. (C) Stimulation interface of the 5-target BCI. Flicker frequencies $f_{1}$-$f_{5}$ were 8–12 Hz for LF band, 18–22 Hz for MF band, 28–32 Hz for HF band, and 38–42 Hz for SHF band. (D) The rTMS paradigm. A total of 1200 pulses were delivered with 24 trains of 10 s at 5 Hz, separated by 5-s intervals.

Phosphene testing was conducted for each participant to determine the optimal rTMS position and stimulation power. Each participant attended two sessions separated by at least 1 week to mitigate carry-over effects. The procedure for both sessions was identical, except for the type of rTMS applied, that is, real rTMS or sham rTMS. EEG recordings were collected during both the SSVEP task and the resting state at three time points: before rTMS stimulation (Pre), immediately after stimulation (Post0), and 20 min after stimulation (Post20). Resting-state EEG was recorded for 2 min with both eyes open. The experimental workflow of each session is illustrated in Figure 1A.

The SSVEP task comprised four blocks, each separated by a 15-s break. Visual stimuli from four frequency bands were presented in randomized order within each block. The frequency bands were defined as follows: LF (8–12 Hz), MF (18–22 Hz), HF (28–32 Hz), and SHF (38–42 Hz). The selected frequency bands were chosen to cover a broad range of stimulation frequencies, from traditionally used low frequencies to more user-friendly high frequencies. This design facilitates a systematic evaluation of how rTMS modulates SSVEP performance across varying conditions. Each frequency band consisted of five 1 Hz spaced targets, which were presented in random order. Each trial included a 0.5-s cue, a 4-s flickering of targets, and a 0.5-s rest. The SSVEP task workflow is illustrated in Figure 1B, and the stimulation interface is shown in Figure 1C.

Two rTMS conditions (real and sham) were pseudorandomized and counterbalanced across participants. The rTMS paradigm is illustrated in Figure 1D. Real rTMS consisted of 24 trains of 10 s at 5 Hz, separated by 5-s intervals, delivering a total of 1200 pulses over 6 min. For sham stimulation, the coil was positioned at a 90° angle to the occiput, producing sensations and noise similar to real rTMS without introducing brain activation. Participants were kept blind to the condition they received. The 5 Hz stimulation frequency was selected based on prior evidence demonstrating its excitatory effects on cortical activity (Lefaucheur et al., 2014). Additionally, recent neurophysiological studies suggest that attention improves neural signal reliability primarily by reducing low-frequency (<5 Hz) correlated variability across neuronal populations, thereby improving the SNR more effectively than merely increasing firing rates (Cohen & Maunsell, 2009; Mitchell et al., 2009).

### 2.3 EEG recording and preprocessing

Participants were seated in an acoustically isolated room with dimmed lighting, facing a 24-inch monitor (aspect ratio: 16:9; refresh rate: 240 Hz). We implemented the user interface using the Psychtoolbox toolbox in MATLAB. A TMS-compatible EEG cap (EASYCAP GmbH, Germany), connected with a 64-channel BrainAmp DC system (BrainProducts Inc., Munich, Germany), was used to acquire EEG data. Electrodes were placed according to the international 10-20 system, with FCz as the reference and AFz as the ground. Impedance was kept below 10 k Ω during recording. EEG signals were collected at a 5 kHz sampling rate, with an online filter at DC to 200 Hz.

EEG was preprocessed in MATLAB using the EEGLAB toolbox. Data were bandpass-filtered between 1 to 90 Hz and down-sampled to 250 Hz. Independent component analysis (ICA) was performed to remove eyeblink-related artifacts using the ADJUST (1.1) plugin.

### 2.4 Transcranial magnetic stimulation

We used a Rapid square Magstim stimulator with a BrainSight TMS navigation system (Magstim Co, Whitland, Dyfed, UK), connected to a figure-of-eight coil (external diameter of each wing, 90 mm).

We first determined the phosphene and motor thresholds through single-pulse TMS. The phosphene threshold was defined as the minimum stimulation intensity that induced phosphenes in at least three out of five trials. The coil was positioned vertically, with its handle pointing upward, and navigated to the left V1. Given that prolonged light deprivation could increase cortical excitability in the occipital cortex (Boroojerdi et al., 2000), a break was taken every 10–12 min, during which participants’ eyes were open and lights were turned on. Stimulation was applied initially at 40% of the stimulator output. The intensity of the stimulation was increased by 5% steps until phosphenes were reported. The threshold was subsequently fine-tuned using 1% increments and decrements (Waterston & Pack, 2010).

Navigated rTMS was performed at the location where the phosphene threshold was measured or at the primary visual cortex (i.e., MNI: [x=−18.6,y=−101.3,z=−10] ) for participants who did not perceive any phosphene. Stimulus output during rTMS was set to the phosphene threshold in participants with phosphene thresholds below 76% of stimulator output or, following published guidelines (R. Chen et al., 1997), to 110% of the motor threshold for those who reported no phosphenes, following previous studies (Fumal et al., 2006; Waterston & Pack, 2010).

### 2.5 Decoding algorithms

We employed two traditional decoding methods for target classification in the SSVEP task. The first one was a calibration-free method named filter-bank canonical correlation analysis (FBCCA) (X. Chen, Wang, Gao, et al., 2015), and its template is artificial sinusoidal signals (${\text{Y}}_{f_{k}},k=1,\;2,\ldots ,\;5$ ). $f_{k}$ is the frequency for the $k^{th}$ target. N band-pass filters are first applied to the original EEG X to obtain sub-band components (${\text{X}}_{SB_{n}},n=1,\;2,\ldots ,N$ ). Then, the canonical correlation analysis is performed on each sub-band component and the template for each target separately. From X. Chen, Wang, Gao, et al. (2015), the feature for target identification is calculated as a weighted sum of the squared correlation values for all sub-band components (i.e., $\rho_{k}^{1},\ldots ,\rho_{k}^{N}$). Specifically, the feature is given by: ${\bar{\rho }}_{k}=\sum_{n=1}^{N}(n^{-1.25}+0.25)$ . The frequency corresponding to the maximum feature is the identification result. This process is repeated for each trial, and the average accuracy across 4 blocks is used as the final results for each subject. The number of filter-banks N is set to 7, 5, 3, and 2 for the LF, MF, HF, and SHF frequency bands, respectively.

The second one was a calibration-based method named task-discriminant component analysis (TDCA) (B. Liu et al., 2021), and its template was obtained by averaging the individual trials according to each target. In our study, each block was tested in turn, and the other three blocks were used to construct the individual template and for spatial filter calculation. The spatial filter was obtained by performing discriminant analysis with respect to all classes, and details can be found in B. Liu et al. (2021). The average accuracy across four blocks was used as the final results for each subject.

For both decoding methods, we introduced the concept of cumulative decoding effect (CDE) as a novel approach to evaluating the decoding performance of SSVEP. Traditional decoding methods typically extract a specific signal segment starting from time zero, such as 1 s for decoding. However, this approach makes it hard to reflect the decoding performance of the entire signal within a BCI system. To address this, we proposed using CDE to assess the overall accuracy improvement over signal durations ranging from 0.5 s to 4 s, thereby capturing performance changes across the entire signal length rather than focusing on differences at a single time point. Specifically, the decoding accuracy was calculated at 0.5 s intervals for signal durations between 0.5 s and 4 s. These accuracy values were then used to construct a curve representing accuracy versus signal length. The ratio of the area under the curve to the total area represents the CDE, with a range of 0–100%.

### 2.6 Statistical analysis

For SSVEP decoding performance, we performed a two-way repeated-measures ANOVA (rm-ANOVA), with Condition (real and sham) and Time (Post0 and Post20) as factors. One-tailed paired t-tests were used to test the significance between real and sham rTMS. Besides, a two-way rm-ANOVA was used for microstate analysis, with Microstate class (A, B, C, and D) and Time (Pre and Post0) as factors.

**Fig. 2.**
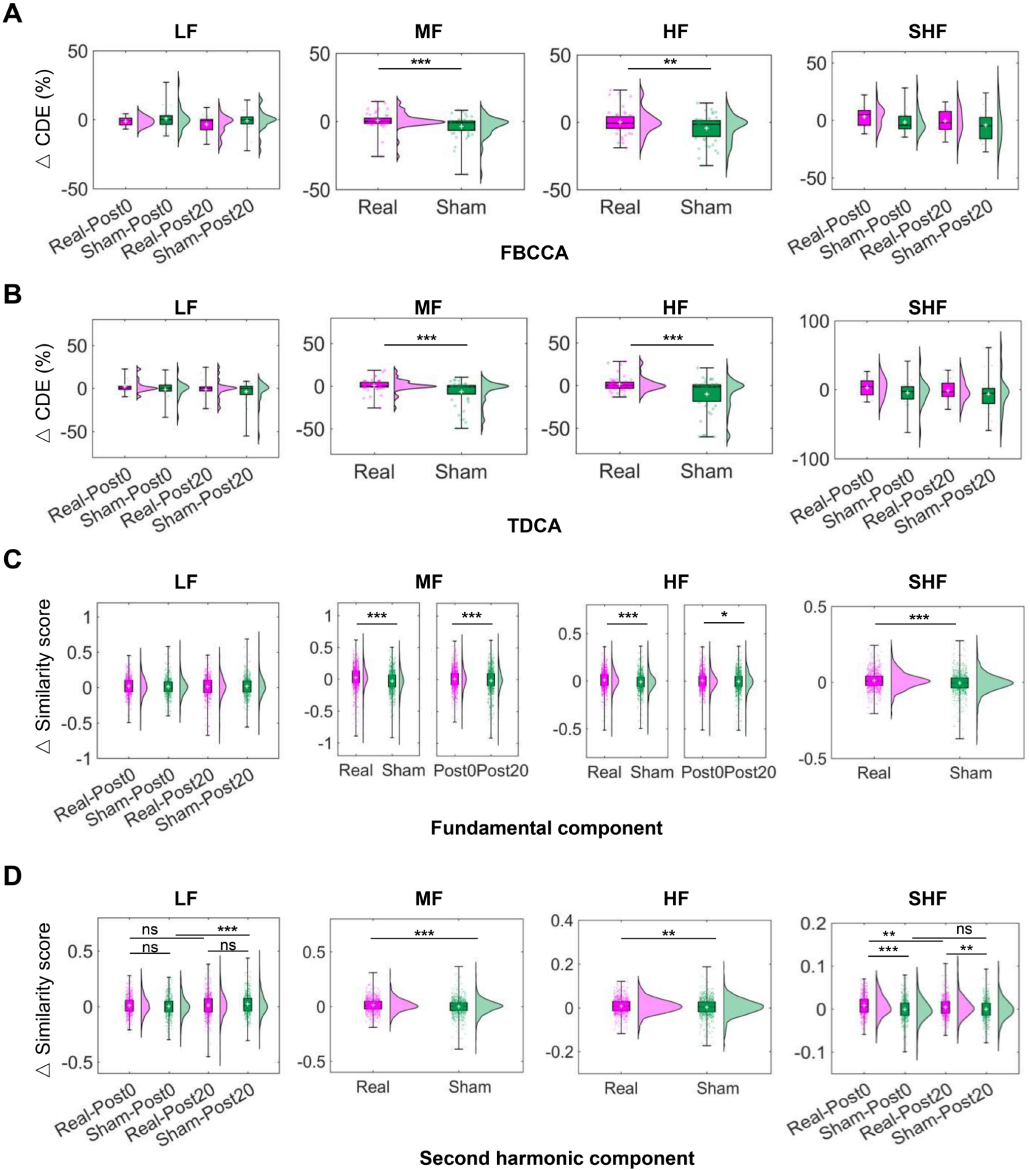
BCI performance improvement and the enhanced features of SSVEP. (A) Changes in CDE using FBCCA. Rm-ANOVA revealed significant main effects of Condition for MF (p<0.01 ) and HF (p<0.05 ). One-tailed paired t-tests showed greater changes under the real condition than the sham for MF (p<0.001 ) and HF (p<0.05 ). (B) Changes in CDE using TDCA. Significant main effects of Condition were observed for MF and HF (both: p<0.01 ), with paired t-tests indicating larger changes under the real condition (both: p<0.001 ). (C) Changes in similarity to fundamental sine–cosine waves. LMM revealed significant Condition effects for MF, HF, and SHF (all: p<0.05 ), and Time effects for MF and HF (both: p<0.05 ). (D) Changes in similarity to second harmonic sine–cosine waves. LMM identified significant Condition×Time interaction effects for LF and SHF (both: p<0.05 ), and Condition effects for MF and HF (both: p<0.05 ). Decoding performance and similarity scores are summarized in Supplemental Tables S5 and S6, respectively.

**Fig. 3.**
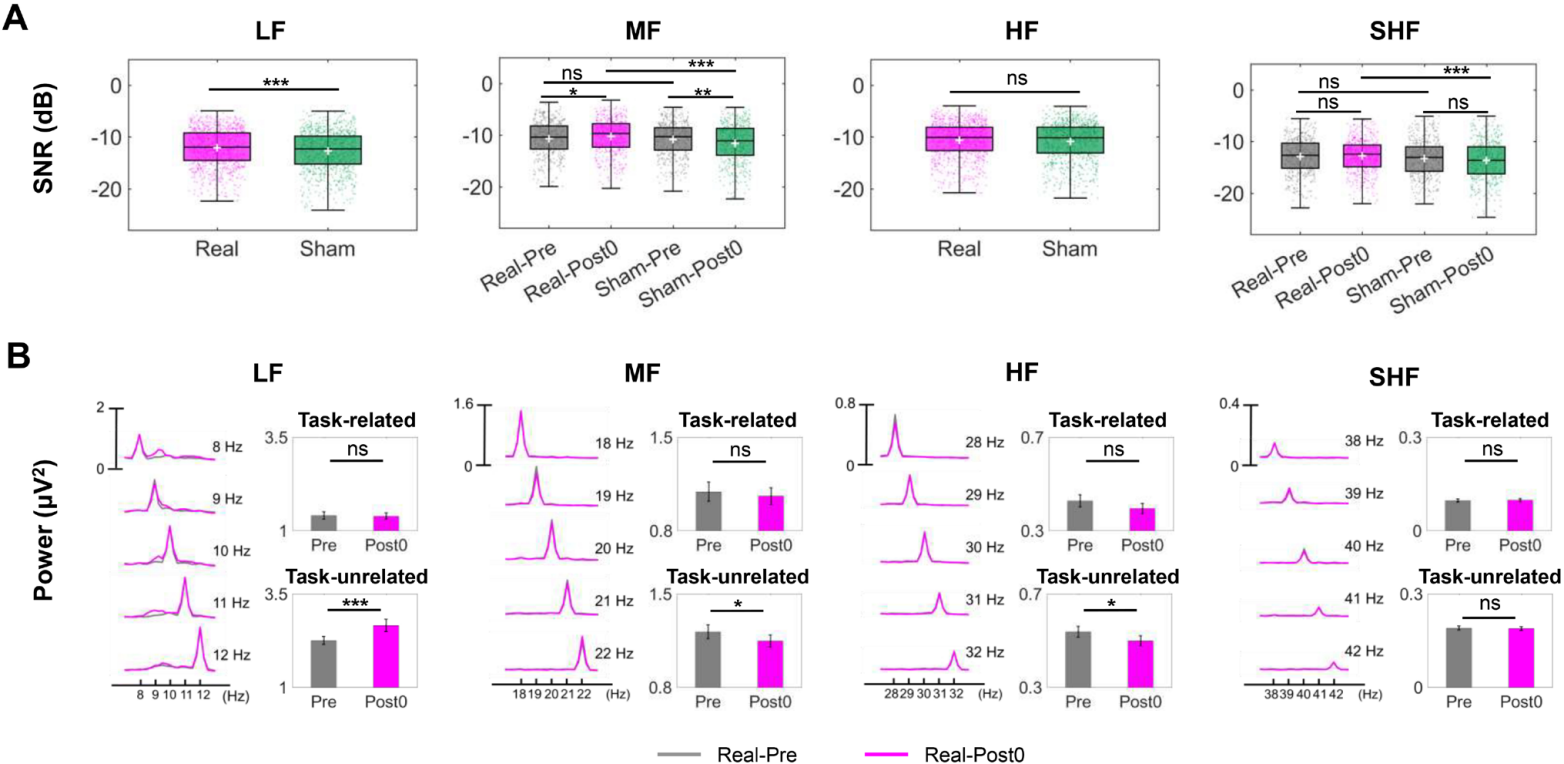
Improvement in SNR and suppression of task-unrelated components in SSVEP. (A) SNR for each band was analyzed using LMM (n=480 for Real-Pre, Real-Post0, Sham-Pre, Sham-Post0). Significant main effect of Condition was found for LF (p<0.001 ), MF (p<0.001 ), and SHF bands (p<0.01 ). Significant Condition × Time interaction effect was found for HF band (p<0.01 ). (B) The power response of each SSVEP target at Pre and Post0. A one-tailed paired t-test (n=480 ) revealed that task-unrelated components were significantly suppressed in the MF and HF bands following real rTMS (both: p<0.05 ), but amplified in the LF band (p<0.001 ). SNR and the power of task-related/unrelated components are summarized in Supplemental Tables S8 and S9, respectively.

The similarity score changes (Fig. 2C, D) and SNR (Fig. 3A) during the SSVEP task were analyzed using a linear mixed-effect model (LMM, Eqs. 1, 2). This model incorporated fixed effects (i.e., Condition and Time) and random effects (i.e., subject-to-subject differences). The model’s fit was compared across conditions using a type III ANOVA test and a one-tailed z-test.

EE∼Condition+Time+Condition :Time+(1|Subject) (1)

Mathematically, this is equivalent to

$$\begin{array}{l} EE_{k}∼\beta_{0}+\beta_{1,i}\cdot Condition_{i}+\beta_{2,j}\cdot Condition_{j}\\ \;\;\;\;+\beta_{3}\cdot (Condition_{i}\cdot Time_{j})+b_{k,i,j}+\epsilon_{k,i,j} \end{array}$$ (2)

where i corresponds to the i-th experimental mode of subject k, and j denotes the experimental time. $b_{k,i,j}$ is the random effect of the subject k of mode i and time j, defined as a Gaussian distribution with a subject-specific mean and variance. $\epsilon_{k,i,j}$ is the error term. Task-related fundamental and harmonic components (Fig. 2C, D) were analyzed by focusing on the after-effects induced by the stimulation. Therefore, in the LMM, the variable ‘Time’ included ‘Post0’ and ‘Post20’, which respectively represent the improvements in features at Post0 and Post20 relative to the Pre. When analyzing the SNR of the SSVEP signal (Fig. 3A), we shifted focus to the absolute value at Pre and Post0. Consequently, in this case, the variable ‘Time’ included ‘Pre’ and ‘Post0’. Statistical significance was determined at p<0.05 following FDR correction, and corrected p-values were reported.

Statistical analysis was performed on EEG power using cluster-based permutation t-tests based on non-parametric statistics in the FieldTrip toolbox (Oostenveld et al., 2011). The significance probability was estimated based on a Monte-Carlo permutation test with a cluster-based correction approach. The permutation test avoids assumptions on the normal distribution of data and solves the problem of multiple comparisons by cluster correction. Initially, the method computed t-values for each sample point in the observed data, applying a threshold defined by the critical t-values corresponding to a probability level of 0.05. A cluster-based correction was then applied, wherein sample points were included in clusters if they had at least one neighboring point with a t-value greater than the critical threshold. The sum of t-values was then assigned to its respective built cluster. This procedure was repeated for 1000 permutations of the data. The significance probability was calculated as the proportion of permuted datasets with larger test statistics than those observed. A p-value below 0.05, that is, > 95% of the permuted datasets did not show clusters with larger sums of t-values, was considered statistically significant.

## 3 Results

### 3.1 rTMS improves target discriminability by enhancing the prominence of both fundamental and harmonic components

Decoding performance was accessed using FBCCA (X. Chen, Wang, Gao, et al., 2015) (Fig. 2A) and TDCA (B. Liu et al., 2021) (Fig. 2B) with 24 participants contributing one sample each. A two-way rm-ANOVA revealed no significant Condition × Time interaction for any frequency band (all: p>0.05 ), but a significant main effect of Condition was observed for MF and HF (both: p<0.05 ) (Supplemental Tables S1 and S2). One-tailed paired t-tests indicated that performance gains under the real condition were significantly greater than those under the sham condition for both MF and HF bands using both FBCCA and TDCA (FBCCA-MF: +4.36%, t(47)=4.026, p<0.001 ; FBCCA-HF: +4.48%, t(47)=2.714, p<0.01 ; TDCA-MF: +7.53%, t(47)=3.893, p<0.001 ; TDCA-HF: +11.47%, t(47) =3.761, p<0.001 ). These results suggest that rTMS effectively enhanced SSVEP decoding performance, specifically for the MF and HF bands.

To further investigate the modulation effects of rTMS on the fundamental and harmonic components of SSVEP separately, we computed the canonical correlation coefficients (similarity scores) between the SSVEP and the periodic waveforms of the fundamental frequency and its second harmonics. Changes in similarity scores were analyzed using an LMM (Supplemental Tables S3 and S4). For the fundamental component (Fig. 2C), no significant interaction effect of Condition × Time was observed for any frequency band (all: p>0.05 ). However, a significant main effect of Condition was identified for MF, HF, and SHF (all: p<0.05 ). One-tailed z-tests revealed that the average change following real rTMS was significantly greater than that observed after sham rTMS for MF (z=7.128,p<0.001 ), HF (z=4.407,p<0.001 ), and SHF (z=5.558,p<0.001 ). Furthermore, a significant main effect of Time was found for MF and HF (both: p<0.05 ), with significantly greater changes at Post0 than Post20 for MF (z=3.250,p<0.001 ) and HF (z=1.793,p<0.05 ). For the second harmonic component (Fig. 2D), a significant interaction effect of Condition × Time was observed for LF and SHF bands (all: p<0.05 ). For LF band, a greater change at Post20 than Post0 under the sham condition was found (z=3.706, p<0.001 ). For the SHF band, the change following real rTMS was significantly greater than that observed after sham rTMS at both Post0 (z=5.912, p<0.001 ) and Post20 (z=3.139, p<0.01 ). Besides, significantly greater changes at Post0 than Post20 were detected under the real condition (z=2.573, p<0.01 ), but not under the sham condition (z=0.433, p>0.05 ). Furthermore, a significant main effect of Condition was found for MF and HF (both: p<0.01 ), with significantly greater changes after real rTMS than sham rTMS for MF (z=4.613, p<0.001 ) and HF (z=2.893, p<0.01 ). The results indicate that, within the MF, HF, and SHF frequency bands, real rTMS enhances both the fundamental and harmonic components compared to sham rTMS.

### 3.2 The enhanced prominence of SSVEP components is achieved by suppressing task-unrelated components rather than amplifying task-related components

We investigated the underlying mechanisms driving the enhancement of the prominence of fundamental components through spectral power analysis. The analysis was conducted based on the occipital 9 channels used for decoding.

The SNR for each trial was analyzed using LMM (Supplemental Table S7) and one-tailed z-tests (Fig. 3A). For LF and HF bands, a significant main effect of Condition was identified (LF:p<0.001; HF:p<0.05 ), with z-tests indicating higher SNR under the real condition (LF: z=3.703, p<0.001 ; HF: z=1.303, p=0.096 ). For MF and SHF bands, significant Condition × Time interaction effects were detected (MF: p<0.001 ; SHF: p<0.05 ). Then, z-tests revealed that the real condition exhibited a significantly higher SNR after stimulation but not at Pre (Real-Post0 vs. Sham-Post0, MF: z=5.437, p<0.001 ; SHF: z=3.852, p<0.001 ; Real-Pre vs. Sham-Pre, MF: z=0.383, p>0.05 ; SHF: z=1.618, p>0.05 ). Besides, for MF band, the SNR significantly increased after real rTMS (Real-Post0 vs. Real-Pre, z=2.309, p<0.05 ) and decreased after sham rTMS (Sham-Post0 vs. Sham-Pre, z=2.857, p<0.01 ).

Next, we separately analyzed task-related components (defined as the power at the stimulation frequency) and task-unrelated components (defined as the power within ±1 Hz of the stimulation frequency) (Fig. 3B). A one-tailed paired t-test (n=480 ) revealed that task-unrelated components were significantly suppressed in the MF and HF bands following real rTMS (both: p<0.05 ), but were amplified in the LF band (p<0.001 ). These findings suggest that the prominence of task components is achieved by suppressing task-unrelated components rather than amplifying task-related components.

### 3.3 rTMS enhanced visual processing network during the task, which can be predicted by cortical excitability measured by PT

Microstate analysis was adopted to assess the spatiotemporal dynamics of large-scale brain activity with sub-second resolution. It captures the temporal sequence and duration of quasi-stable brain states, making it well-suited for evaluating rTMS-induced modulation during both task and resting states. Given its established correspondence with large-scale functional networks identified by fMRI (Britz et al., 2010; Musso et al., 2010), microstate analysis provides an informative and globally interpretable framework for assessing network-level brain dynamics.

**Fig. 4.**
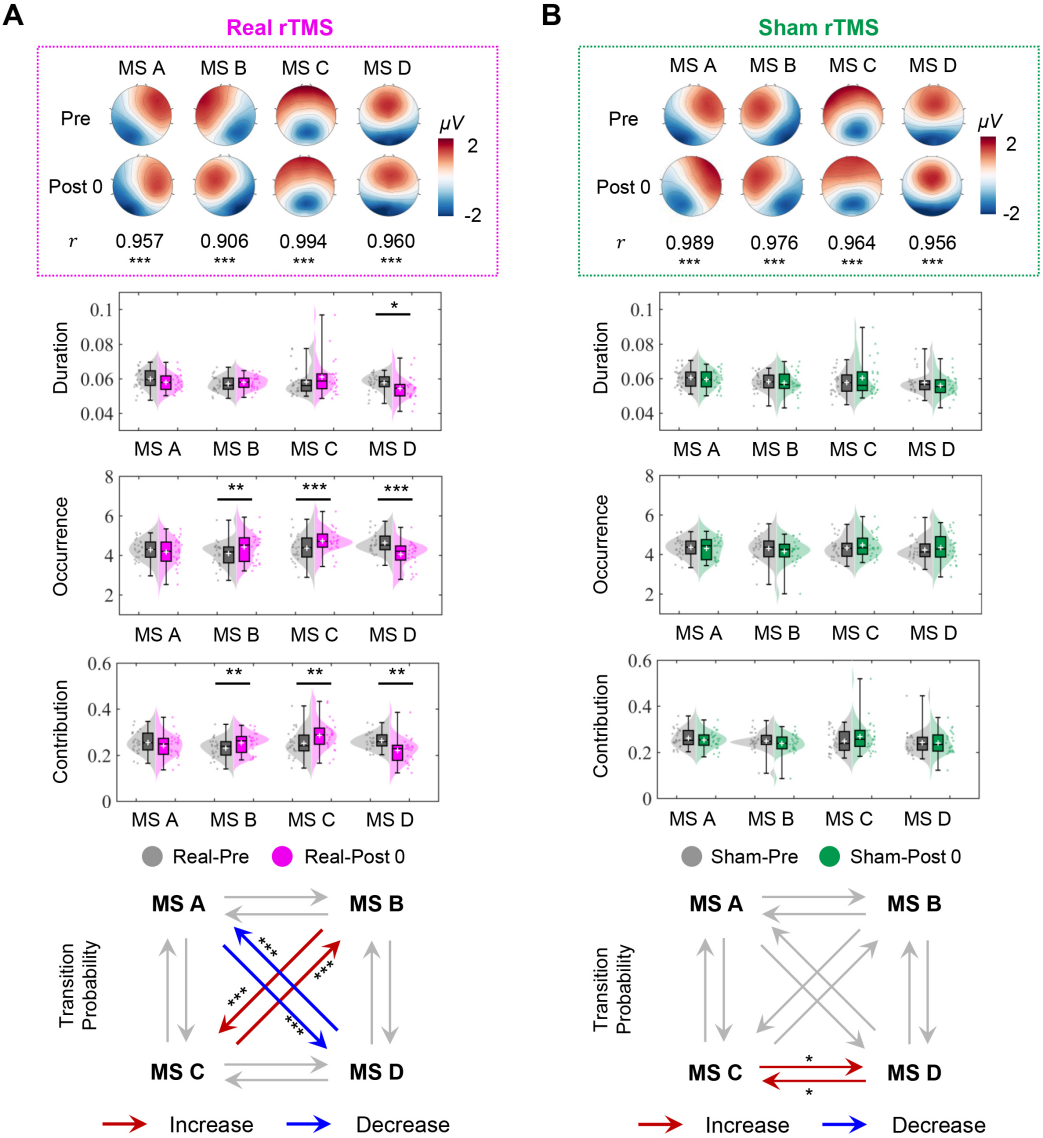
Microstate analyses results for SSVEP tasks at Pre and Post0 under real and sham conditions. (A) Microstate configurations under the real condition. (B) Microstate configuration under the sham condition. Four microstate classes (A-D) explained 70.64%, 72.96%, 72.33%, and 73.45% of the global variance for the Real-Pre and Real-Post0, Sham-Pre, and Sham-Post0, respectively. A two-way rm-ANOVA found significant Time × Microstate Class interaction for the duration, occurrence, and contribution under the real condition. One-tailed paired t-tests (n=24 ) detected a significant increase in occurrence and contribution for microstates B and C, and a significant decrease in duration, occurrence, and contribution was detected for microstate D. Real rTMS also increased transitions between A and D and decreased those between B and C (p<0.001 ), while sham rTMS increased transitions between C and D (p<0.05 ).

Microstate dynamics during the SSVEP task were analyzed for Pre and Post0 under real and sham conditions. The topographies of the four microstate classes (Fig. 4A, B), consistently reported in Michel and Koenig (2018), explained 70.64%, 72.96%, 72.33%, and 73.45% of the global explained variance for the Real-Pre and Real-Post0, Sham-Pre, and Sham-Post0, respectively. Spatial correlations between Pre and Post0 of microstate A-D are 0.957, 0.906, 0.994, and 0.960 under the real condition, and 0.989, 0.976, 0.964, and 0.956 under the sham condition (all: p<0.001 ). These indicated that the largest difference occurred in microstate B under the real condition (r(62)=0.906,p<0.001 ).

Mean duration, occurrence, and contribution were computed for each participant, and a two-way rm-ANOVA (Supplemental Table S10) revealed a significant Time × Microstate Class interaction for all three metrics under the real condition (all: p<0.05 ). One-tailed paired t-tests (n=24 ) indicated that, after real rTMS, microstate B had a significantly increased occurrence (t(23)=3.239,  p<0.01 ) and contribution (t(23)=2.738, p<0.01 ). Microstate C had a significantly increased occurrence (t(23)= 4.250, p<0.001 ) and contribution (t(23)=3.242, p<0.01 ). Microstate D had a significantly decreased mean duration (t(23)=2.876, p=0.05 ), occurrence (t(23)=4.191,  p<0.001 ), and contribution (t(23)=3.724, p<0.01 ). The transition probabilities between microstates are shown in the bottom row of Figure 4 for both the real and sham stimulation conditions. Following real rTMS stimulation, the bidirectional transition probabilities between microstates A and D increased significantly (both: p<0.001 ), whereas those between microstates B and C decreased significantly (p<0.001 ). In contrast, under the sham condition, a significant increase in the transition probabilities between microstates C and D was observed (both: p<0.05 ).

Microstate analysis of resting-state EEG was also conducted, with results shown in Supplemental Figure S1 and Supplemental Table S11. The microstates C and D exhibit consistent changes between the resting state and the SSVEP task state.

**Fig. 5.**
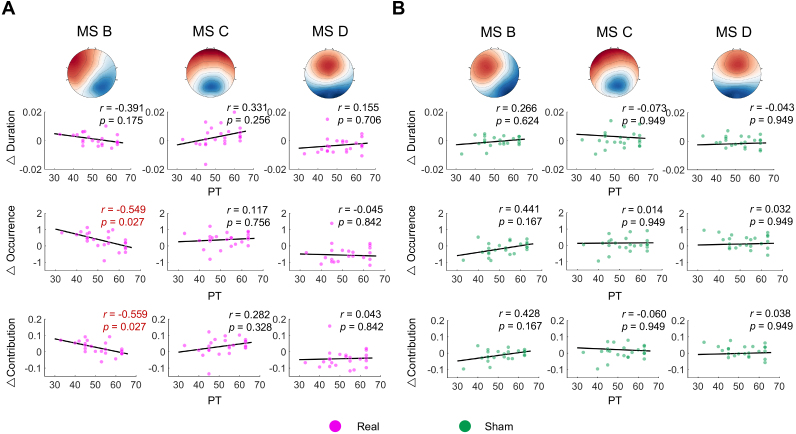
The relationship between the PT and change of microstate B, C, and D metrics (i.e., duration, occurrence, and contribution). Pearson correlation analysis was performed (n=24 ). (A) The real condition. (B) The sham condition. Significant correlations after FDR correction are highlighted in red.

Pearson correlation analyses (Fig. 5A) revealed a significant negative correlation between PT (as lower PT reflects higher cortical excitation) and changes in the occurrence and contribution of microstate B (occurrence: r(24)=−0.549, p=0.027 ; contribution: r(24)=−0.559,p= 0.027 ), highlighting the dominant effect of rTMS on microstate B, which is associated with visual processing. This association suggests that higher cortical excitability leads to greater changes in the visual network induced by rTMS.

### 3.4 rTMS-induced changes in resting-state power are correlated with visual network dynamics during task state

**Fig. 6.**
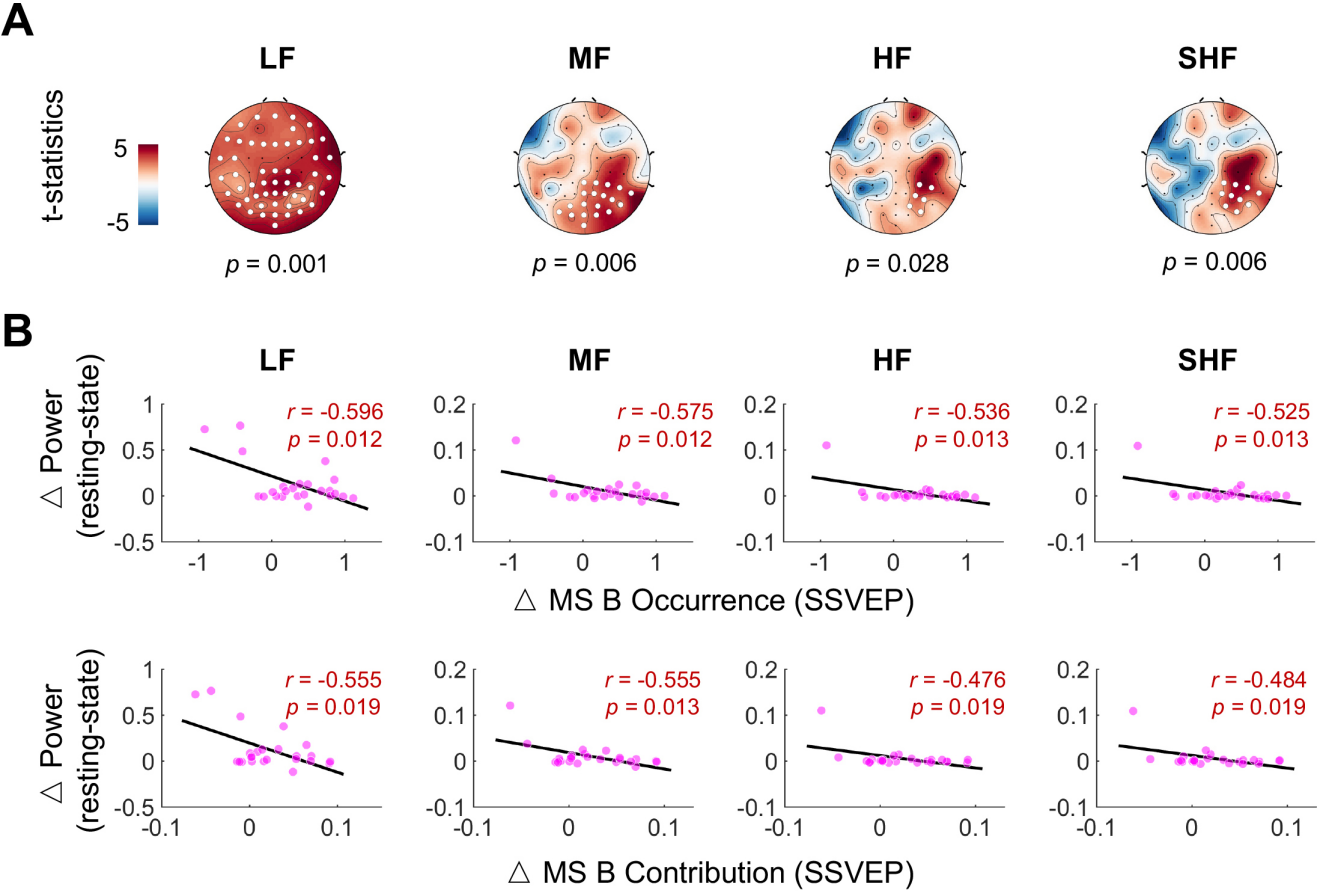
Correlation between visual network changing in SSVEP task and resting-state power changing under the real condition. (A) Significant spatial clusters were identified using a non-parametric cluster test with 1000 permutations (one-tail t-test) comparing Pre with Post0. Significant clusters are denoted by white circles over the electrodes. Positive clusters were observed for all frequency bands under the real condition. (B) Pearson correlation analysis (n=24 ) revealed significant moderate to strong negative correlations between changes in microstate B metrics (occurrence and contribution) during SSVEP tasks and changes in EEG power across all four frequency bands in the resting state (all: p<0.05 ).

Resting-state EEG power corresponding to four frequency bands during the SSVEP task was calculated. Significant spatial clusters were identified using cluster-based permutation t-tests (Fig. 6A). Under the real condition, significant positive clusters were observed across the whole brain for LF and in the right parietal and occipital region for MF, HF, and SHF (all: p<0.05 ).

Based on the evidence that rTMS predominantly affects microstate B occurrence and contribution during the SSVEP task, we further explored the correlation between these indicators and the energy features in the resting state (Fig. 6B). A significant negative correlation was found between the change of occurrence and the change of each band power (LF: r(24)=−0.596, p=0.012 ; MF: r(24)=−0.575, p=0.012 ; HF: r(24)=−0.536, p=0.013 ; SHF: r(24)=−0.525, p=0.013 ), and between the change of contribution and the change of each band power (LF: r(24)=−0.555, p=0.019 ; MF: r(24)=−0.555, p= 0.013 ; HF:  r(24)=−0.476, p=0.019 ; SHF: r(24)=−0.484,  p=0.019 ).

### 3.5 Resting-state power before RTMS predicts SSVEP enhancement in the corresponding band

**Fig. 7.**
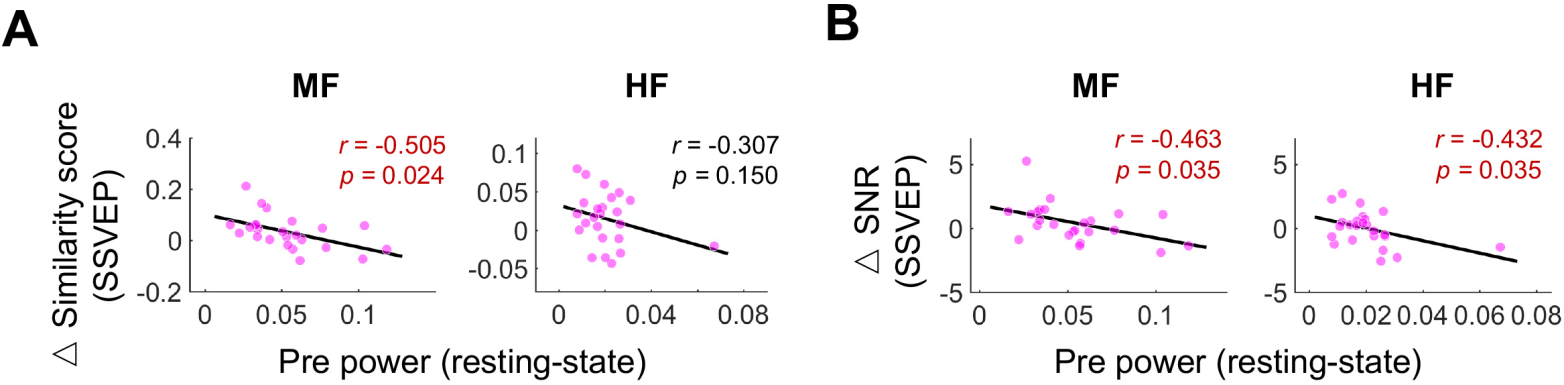
Correlation between resting-state power before rTMS and changes in SSVEP prominence within corresponding frequency bands under the real condition. (A) Pearson correlation analysis (n=24 ) between baseline resting-state power in the resting state and changes in similarity score (combined fundamental and the second harmonic components) during the SSVEP task within corresponding frequency bands. A significant moderately negative correlation was found in the MF band (p<0.05 ). (B) The relationship between baseline resting-state power and changes in wide SNR during the SSVEP task within corresponding frequency bands. A significant moderately negative correlation was detected in the MF and HF bands (both: p<0.05 ). Significant correlations after FDR correction are highlighted in red.

Relationships between resting-state power before stimulation and changes in similarity scores with fundamental and second harmonic waves (Fig. 7A) and wide SNR during the SSVEP task (Fig. 7B) were examined. A significant negative correlation was found between resting-state power in the MF band and changes in similarity scores (r(24)=−0.505, p=0.024 ). Significant negative correlations were detected between changes in wide SNR and resting-state power in the MF (r(24)=−0.463,  p=0.035 ) and HF (r(24)=−0.432, p=0.035 ) bands.

### 3.6 Decoding performance enhancement is consistent in deep learning-based methods

Deep learning methods have demonstrated outstanding performance in SSVEP decoding and are considered a key direction for future BCI development. To examine whether the rTMS-induced improvements observed in traditional methods extend to deep learning-based approaches, we evaluated decoding performance before and after rTMS using three representative models:

- Compact-CNN (Waytowich et al., 2018): A subject-specific model constructed based on CNN.
- Conv-CA (Y. Li et al., 2020): A subject-specific model using a two-branch structure.
- ConsenNet (X. Zhang et al., 2024): A cross-subject pretraining model that uses frequency-domain input obtained via FFT, followed by subject-specific fine-tuning.

**Fig. 8.**
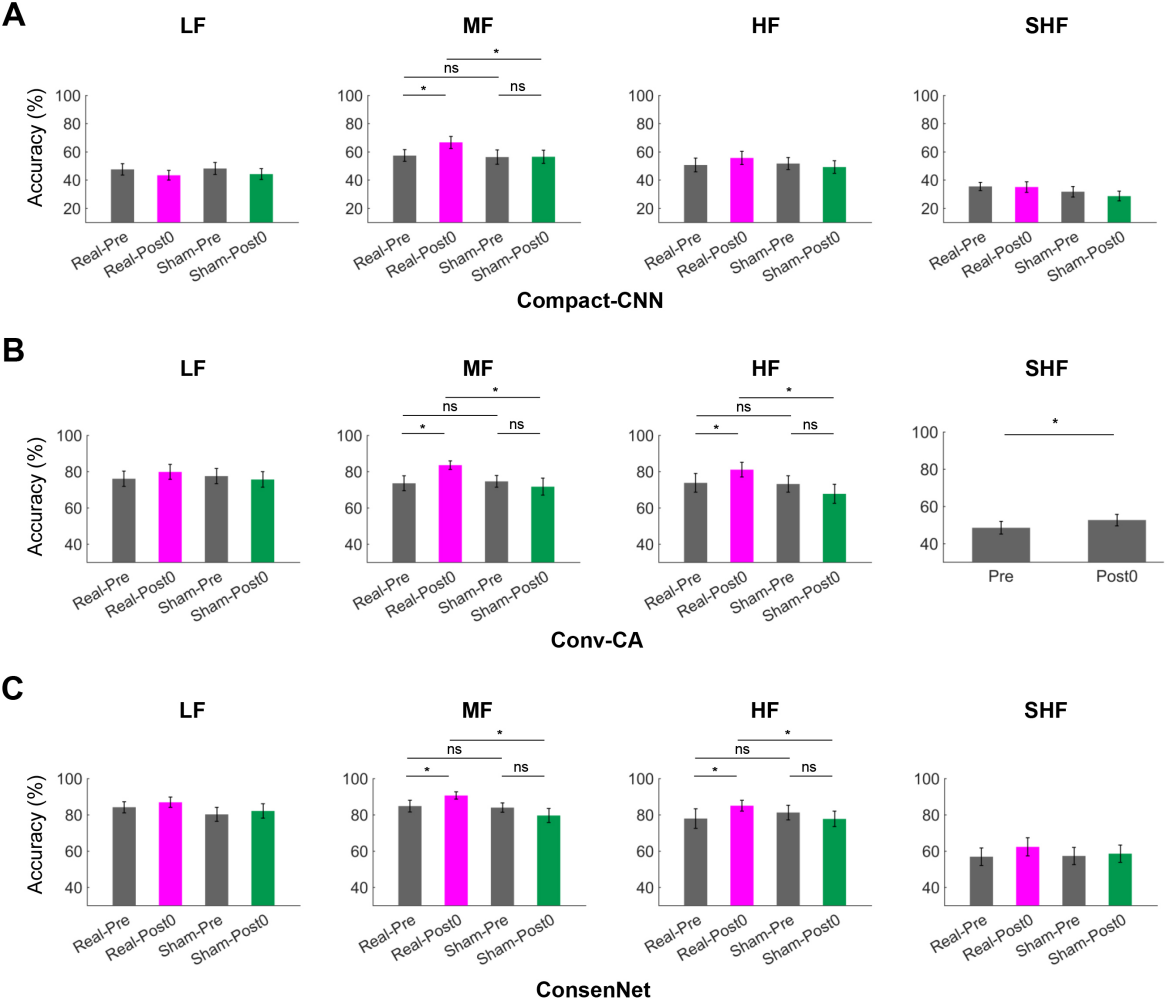
Decoding performance of deep learning methods before and after rTMS. (A) Compact-CNN results. (B) Conv-CA results. (C) ConsenNet results. For the MF band, significant Time × Condition interactions were observed across all three methods. One-tailed paired t-tests showed that decoding accuracy significantly increased in Real-Post0 compared to both Real-Pre and Sham-Post0 (p<0.05 ). For the HF band, significant Time × Condition interactions were found for Conv-CA and ConsenNet, with decoding accuracy significantly higher in Real-Post0 than in both Real-Pre and Sham-Post0 (p<0.05 ). For the SHF band, Conv-CA showed a significant main effect of Time (p<0.05 ), with significantly higher decoding accuracy after stimulation than before (p<0.05 ). A full summary of decoding accuracy is presented in Supplemental Table S15.

For Compact-CNN and Conv-CA, subject-specific training and testing were conducted using raw time-domain EEG signals. For each subject and each time point, a leave-one-block-out strategy was employed. The average decoding accuracy across the four blocks was taken as the final result for that time point. For ConsenNet, each subject was treated in turn as the “new subject”, with data from the remaining 23 subjects used for pretraining. The EEG signals were transformed into the frequency domain via FFT before being input into the model. Fine-tuning was performed on the calibration data of the target subject, using a leave-one-block-out strategy. Decoding results for all models are presented in Figure 8.

Rm-ANOVA results for Compact-CNN, Conv-CA, and ConsenNet are presented in Supplemental Tables S12, S13, and S14, respectively. For the MF band, significant Time × Condition interactions were observed across all three methods. One-tailed paired t-tests showed that decoding accuracy significantly increased in Real-Post0 compared to both Real-Pre (+9.375% for Compact-CNN, +10.000% for ConvCA, and +5.833% for ConsenNet) and Sham-Post0 (+10.209%, +11.875%, and +11.042%, respectively) (all: p<0.05 ). For the HF band, significant Time × Condition interactions were found for Conv-CA and ConsenNet. Decoding accuracy was significantly higher in Real-Post0 than in both Real-Pre (+7.292% for Conv-CA and +7.083% for ConsenNet) and Sham-Post0 (+12.292% and +7.292%) (all: p<0.05 ). For the SHF band, Conv-CA showed a significant main effect of Time (p<0.05 ), with decoding accuracy increasing by 3.75% after stimulation.

These consistent improvements across diverse decoding architectures confirmed that rTMS enhanced SSVEP decoding performance, highlighting the robustness and generalizability of its neuromodulatory effects.

## 4 Discussion

This study demonstrates that 5 Hz rTMS applied to the primary visual cortex can enhance the performance of SSVEP-based BCI, particularly within mid-high frequency bands. Quantitative analysis of brain responses to 5 Hz rTMS revealed that these performance gains were driven by rTMS-induced modulation of the relative proportion of task-related and task-unrelated components. These results support our hypothesis that rTMS induces decoding performance by enhancing EEG signal SNR. Based on our findings and prior research, we propose that this effect is mediated by the influence of 5 Hz rTMS on visual attention.

An increased occurrence and contribution of microstate B were observed during SSVEP tasks following 5 Hz rTMS, indicating enhanced functioning of the visual network (Bréchet et al., 2019; Custo et al., 2017), which encompasses visual processing and visual-spatial attention (J. Liu et al., 2020; Lu et al., 2025; Zappasodi et al., 2019). Notably, this enhancement of microstate B was primarily evident in the task state, with its changes negatively correlated with alterations in resting-state power (Fig. 5). The change of the visual network in the SSVEP task may be due to the brain activate a task-specific network only when the task is performed (Huang, 2019). Previous studies have suggested that the brain’s functional network architecture during task performance is primarily shaped by intrinsic network dynamics, which are also present during rest, and secondarily by task-general and task-specific network changes (Cole et al., 2014). This large-scale mechanism of task-evoked activity flow over the intrinsic network dynamics (Cole et al., 2016) is underlying the closely associated activity between resting-state and task-state (Cole et al., 2014; Y. Zhang et al., 2013). Here, microstates C and D exhibited consistent changes across both task and resting states. Microstate C, which partially overlaps with the default mode network (Bréchet et al., 2019; Custo et al., 2017), showed a significant increase in both states, with a marginal correlation (r(24)=0.354, p=0.089 ). This finding supports the notion that intrinsic brain dynamics link resting and task states. Given that the SSVEP task primarily involves simple visual perception with minimal engagement of higher-order cognitive functions, the opposite changes observed in microstate D may represent a compensatory mechanism to maintain brain functioning, in agreement with observations from previous studies (Y.-N. Li et al., 2024; Yao et al., 2024). To explore whether individual differences in cortical excitability influence behavioral performance, we examined the correlation between phosphene thresholds (PTs) and decoding accuracy in the Real condition at each time point (Pre, Post0, and Post20), as well as the performance improvement (Post0–Pre). No significant correlations were found. One possible explanation is that decoding accuracy reflects an aggregated measure across trials and channels, which may obscure trial-specific neural modulations associated with PTs. In contrast, EEG microstate metrics and spectral features offer more temporally resolved and fine-grained assessments of brain activity, potentially making them more sensitive to individual variability in excitability.

SSVEPs are photic-driving responses that elicit brain activity at both the stimulation and harmonic frequencies (Herrmann, 2001; Wang et al., 2016). Brain responses at the fundamental and harmonic frequencies, typically measured by amplitude or power, are considered task-related components, while responses at other frequencies are regarded as task-unrelated components (X. Chen, Wang, Nakanishi, et al., 2015). The relative proportion of task-related to task-unrelated components, often defined by SNR, determines the discriminability between targets. A higher proportion of task-related components typically improves discriminability. In this study, the improved discriminability induced by 5 Hz rTMS was attributed to the suppression of task-unrelated components, thereby enhancing the SNR. Previous studies investigating neuromodulatory effects over the visual cortex are summarized in Table 1. Investigations into rTMS modulation of V1 using VEPs to assess task-related components (Fumal et al., 2003; Kolbe et al., 2020) have generally reported no significant changes, consistent with our findings. VEPs, as brief responses to intermittent visual stimuli, tend to exhibit low amplitudes easily masked by background EEG activity. Moreover, task-related components in VEPs are typically analyzed by averaging multiple trials, while task-unrelated components remain challenging to quantify. In contrast, SSVEPs, being continuous and periodic signals, enable simultaneous measurement of task-related and task-unrelated components. Our finding of suppressed task-unrelated components may reflect modulation of neuronal firing rates (Waterston & Pack, 2010) and a lasting reduction in noise correlations within V1 (Gutnisky & Dragoi, 2008) induced by 5 Hz rTMS, thereby enhancing population coding accuracy (Gutnisky & Dragoi, 2008). This study provides the first evidence that rTMS can enhance SSVEP-BCI performance by improving signal quality through suppression of background noise.

**Table 1.** Comparative summary of neuromodulation studies on visual evoked responses.

Study	Task	Neuromodulation	Target region	N	Experimental design	Main findings	Effect duration
Fumal et al. (2003)	VEP	1/10 Hz rTMS 900 pulses × 3 sessions	V1	10	Within-subject	1 Hz rTMS reduced N1-P1 and P1-N2 amplitudes and habituation; no effect for 10 Hz.	33 min
Antal et al. (2004)	VEP	Anodal/Cathodal tDCS 1 mA, 15 min	Oz	20	Within-subject	Cathodal decreased, anodal increased N70 amplitude.	Anodal: 15 min Cathodal: 10 min
B. Liu et al. (2017)	SSVEP (6-15 Hz)	Anodal/Cathodal tDCS 1 mA, 15 min	PO7-PO8	12	Sham-controlled within-subject	Both tDCS polarities reduced 7 Hz power; anodal tDCS increased 10 Hz power.	N/A
Kolbe et al. (2020)	VEP	10 Hz rTMS 900 pulses	V1	18	Sham-controlled within-subject	No significant changes in amplitude or latency.	N/A
S. Zhang et al. (2023)	SSVEP (9-11 Hz)	Anodal-tDCS 2 mA, 21 min	Oz	11	Sham-controlled (fixed order) within-subject	Increased amplitude and accuracy (<5%).	N/A
S. Zhang et al. (2024)	SSVEP (9-11 Hz)	Anodal-tDCS 2 mA, 21 min × 5 sessions	Oz	13	Sham-controlled within-subject	Increased amplitude and accuracies (<5%).	N/A
Ours	SSVEP (8-42 Hz)	5 Hz rTMS 1200 pulses	Navigated V1	24	Sham-controlled within-subject	Enhanced visual attention, increased SNR and accuracy (∼10%).	≥20 min

As illustrated in Figure 2, the modulation effects of rTMS varied across frequency bands. Notably, the LF band showed no significant improvements in either decoding accuracy (Fig. 2A and 2B) or task-related feature strength (Fig. 2C and 2D). This result may be attributed to the overlap between the LF band (8–12 Hz) and the endogenous alpha rhythm, which is closely associated with visual attention (Peylo et al., 2021). As shown in Figure 3B, rTMS significantly increased alpha-band power, leading to elevated background activity unrelated to the SSVEP task. This enhancement of task-unrelated components reduced the overall SNR, thereby attenuating the potential benefits of rTMS modulation in this band. Consistent effects were also observed in the resting state (Fig. 6A, LF), further supporting this interpretation. Moreover, both the MF and HF bands exhibited statistically significant improvements in decoding performance following rTMS. Although the SHF band showed a comparable trend in task-related feature enhancement (Fig. 2C, D), its decoding accuracy improvement did not reach statistical significance (Fig. 2A, B). Two factors may account for this discrepancy: (1) Intrinsically weak SSVEP responses at SHF. SSVEP responses in the SHF band are intrinsically weaker (Fig. 3B), resulting in lower SNR and reduced target separability compared with MF and HF. Since rTMS enhances decoding performance by increasing the prominence of task-related components, this low baseline signal strength limits the effectiveness of modulation. As shown in Supplemental Table S6, the relative changes between Real and Sham conditions after TMS were 0.061, 0.023, and 0.018 for MF, HF, and SHF bands, respectively, indicating a clear decrease in modulation magnitude with increasing frequency. Thus, even though rTMS enhanced task-related components at SHF, the absolute signal strength remained insufficient for reliable classification; (2) Fewer usable harmonics at SHF. Human EEG activity is widely considered meaningful below 100 Hz (Singh & Krishnan, 2023), with stable spectrum in this range across large populations (Grummett et al., 2014). The number of harmonics available within this range decreases with increasing stimulation frequency: MF, HF, and SHF bands have approximately 5, 3, and 2 harmonics, respectively. Since SSVEP decoding algorithms rely on these harmonic components to extract discriminative features, fewer harmonics at SHF reduce the amount of usable information.

Lower PT is indicative of higher cortical excitability, as shown in previous studies (Antal et al., 2003; Boroojerdi et al., 2000; Gothe et al., 2002). In the present study, we observed a negative correlation between PT and the occurrence and contribution of microstate B (Fig. 5), suggesting that higher cortical excitability predicts more pronounced rTMS-induced modulation of the visual network. This is consistent with evidence indicating that underlying cortical excitability can influence the rTMS effect (Fierro et al., 2005). Additionally, resting-state power before rTMS was predictive of rTMS-induced changes during the SSVEP task (Fig. 7), potentially reflecting the heightened responsiveness of hypometabolic regions to high-frequency rTMS (Eschweiler et al., 2000). These findings underscore the role of individual neurophysiological variability in determining the efficacy of neuromodulation. Future studies should explore how adaptive rTMS protocols that tailor stimulation parameters to individual neurophysiological profiles to optimize intervention benefits.

The effects of 5 Hz rTMS on decoding performance were sustained over time, with no significant decline relative to sham stimulation. At the signal feature level, task-related components across the MF, HF, and SHF bands were significantly enhanced following rTMS. Although partial attenuation was observed—specifically, a reduction in fundamental components in the MF and HF bands, and in second harmonic components in the SHF band after 20 min—the overall SSVEP signal quality and decoding performance remained superior to both baseline and sham conditions. These findings suggest that the neuromodulatory effects of 5 Hz rTMS can persist for at least 20 min post-stimulation.

To enhance internal validity and control inter-group variability, identical participants were tested in both real and sham rTMS conditions. Our study was conducted on young, healthy participants to ensure controlled experimental conditions. However, as the ultimate goal of BCIs is to restore function or support rehabilitation in individuals with neurological or functional impairments (D. Zhang et al., 2024), future studies should extend to more diverse clinical populations. The observed enhancement of SSVEP decoding performance in mid-to-high frequency bands is particularly relevant for clinical users, as these frequencies improve visual comfort and user tolerance—critical factors for long-term applicability. By improving signal quality and reducing the reliance on extensive training, the proposed rTMS-augmented approach may enhance the accessibility and usability of BCIs for patients with motor or cognitive deficits. Moreover, the non-invasive, short-session 5 Hz rTMS protocol can serve as a pre-use priming intervention, seamlessly integrated into existing BCI workflows. With continuing advances in miniaturized and wearable TMS devices (Qi et al., 2025), the feasibility of deploying rTMS-enhanced BCIs in real-world rehabilitation settings is increasing. Additionally, although our study employed a single session of 5 Hz rTMS and observed sustained improvements in decoding performance over a 20-min period, future studies should explore multi-session stimulation protocols. Prior studies have shown that repeated rTMS sessions can lead to cumulative and longer-lasting effects on cortical excitability and cognitive function (Fumal et al., 2006; Guse et al., 2010; S. Zhang et al., 2024). Such protocols may further prolong or amplify the observed benefits, thereby enhancing the practical applicability of rTMS-augmented BCI systems.

## 5 Conclusion

In conclusion, our findings provide compelling evidence that neuromodulation of V1 significantly enhances SSVEP-based BCI performance, particularly within the MF and HF bands. We further explored the underlying neurological changes induced by both rTMS and sham conditions, offering a comprehensive framework for understanding the mechanisms responsible for these effects. Analysis of EEGs during the SSVEP task revealed that rTMS neuromodulation leads to significant suppression of task-unrelated components, thereby enhancing the SNR of the signal. Moreover, alterations in the configuration of the visual processing network, accompanied by a pronounced increase in alpha power, provide critical evidence that the enhancement of task-related components is mediated by an augmentation of visual attention. This study highlights the potential of rTMS as a neuromodulatory tool for optimizing BCI performance, particularly in terms of attentional enhancement.

## Supplementary Material

Supplementary Material

## Data Availability

The data and code that support the findings of this study are available from the corresponding author upon reasonable request.
