# Supplementary material for: Enhancing visual brain-computer interface through V1-targeted RTMS by modulating visual attention
Source: Imaging Neurosci (Camb). 2025 Nov 17;3:IMAG.a.1013. doi: 10.1162/IMAG.a.1013 (PMC12624365; doi:10.1162/IMAG.a.1013)
Supplement: Supplementary Material [file IMAG.a.1013_supp.pdf]

Supplementary Material

Table S1: Rm-ANOVA results for CDE-FBCCA method in four frequency bands

	**Main effect: Time**	**Main effect: Condition**	**Interaction: Time×Condition**
LF	$F(1, 23) = 2.503, p = 0.127$	$F(1, 23) = 1.374, p = 0.253$	$F(1, 23) = 0.248, p = 0.623$
MF	$F(1, 23) = 3.288, p = 0.083$	$F(1, 23) = 9.415, p = 0.005$	$F(1, 23) = 0.936, p = 0.343$
HF	$F(1, 23) = 3.170, p = 0.088$	$F(1, 23) = 4.759, p = 0.040$	$F(1, 23) = 1.042, p = 0.318$
SHF	$F(1, 23) = 3.764, p = 0.065$	$F(1, 23) = 2.698, p = 0.114$	$F(1, 23) = 0.157, p = 0.696$

Table S2: Rm-ANOVA results for CDE-TDCA method in four frequency bands

	**Main effect: Time**	**Main effect: Condition**	**Interaction: Time×Condition**
LF	$F(1, 23) = 2.858, p = 0.104$	$F(1, 23) = 0.887, p = 0.356$	$F(1, 23) = 0.001, p = 0.977$
MF	$F(1, 23) = 0.207, p = 0.654$	$F(1, 23) = 10.484, p = 0.004$	$F(1, 23) = 3.308, p = 0.082$
HF	$F(1, 23) = 0.728, p = 0.403$	$F(1, 23) = 8.500, p = 0.008$	$F(1, 23) = 0.005, p = 0.947$
SHF	$F(1, 23) = 1.681, p = 0.208$	$F(1, 23) = 1.871, p = 0.185$	$F(1, 23) = 0.467, p = 0.501$

Table S3: The LMM results for fundamental components in four frequency bands

	**Main effect: Time**	**Main effect: Condition**	**Interaction: Time×Condition**
LF	$F(1, 1916) = 1.416, p = 0.234$	$F(1, 1916) = 0.945, p = 0.331$	$F(1, 1916) = 1.359, p = 0.238$
MF	$F(1, 1916) = 10.38, p = 0.001$	$F(1, 1916) = 38.71, p = 6.025\text{E}{-10}$	$F(1, 1916) = 0.866, p = 0.352$
HF	$F(1, 1916) = 4.224, p = 0.040$	$F(1, 1916) = 15.93, p = 6.853\text{E}{-5}$	$F(1, 1916) = 1.033, p = 0.310$
SHF	$F(1, 1916) = 3.343, p = 0.068$	$F(1, 1916) = 20.395, p = 6.682\text{E}{-6}$	$F(1, 1916) = 0.309, p = 0.579$

Table S4: The LMM results for second harmonic components in four frequency bands

	**Main effect: Time**	**Main effect: Condition**	**Interaction: Time×Condition**
LF	$F(1,1916) = 0.491, p = 0.484$	$F(1,1916) = 2.429, p = 0.119$	$F(1,1916) = 4.897, p = 0.027$
MF	$F(1,1916) = 1.285, p = 0.257$	$F(1,1916) = 9.491, p = 0.002$	$F(1,1916) = 0.251, p = 0.616$
HF	$F(1,1916) = 0.365, p = 0.546$	$F(1,1916) = 11.667, p = 6.496\text{E}{-4}$	$F(1,1916) = 3.434, p = 0.064$
SHF	$F(1,1916) = 6.716, p = 0.010$	$F(1,1916) = 38.644, p = 9.627\text{E}{-3}$	$F(1,1916) = 4.644, p = 0.031$

Table S5: CDE values with FBCCA and TDCA methods across four frequency bands (mean ± SE %)

		**Real**			**Sham**		
Method	Band	Pre	Post0	Post20	Pre	Post0	Post20
FBCCA	LF	92.068 ± 1.403	90.952 ± 1.557	88.810 ± 2.049	87.411 ± 2.292	88.006 ± 1.912	86.771 ± 2.505
	MF	93.780 ± 1.634	95.729 ± 1.127	93.214 ± 1.699	94.122 ± 1.140	91.086 ± 1.963	89.747 ± 2.322
	HF	83.289 ± 3.172	84.211 ± 2.599	82.530 ± 3.142	82.440 ± 3.077	80.045 ± 3.504	76.042 ± 4.091
	SHF	66.042 ± 4.028	69.286 ± 4.109	65.580 ± 3.891	61.949 ± 4.241	60.283 ± 4.129	57.619 ± 4.358
TDCA	LF	92.470 ± 2.136	94.405 ± 1.363	91.429 ± 2.484	90.938 ± 2.491	89.955 ± 2.864	86.890 ± 4.281
	MF	93.929 ± 1.672	96.161 ± 1.388	92.976 ± 2.240	94.866 ± 1.623	87.024 ± 3.758	88.929 ± 3.044
	HF	88.765 ± 3.518	90.774 ± 2.734	89.375 ± 2.781	91.786 ± 2.029	82.455 ± 4.384	80.804 ± 5.043
	SHF	73.125 ± 3.916	76.339 ± 3.783	71.771 ± 4.058	72.247 ± 4.412	67.961 ± 5.363	66.265 ± 5.083

Table S6: Similarity scores of fundamental and harmonic components across four frequency bands (mean ± SE)

		**Real**			**Sham**		
Component	Band	Pre	Post0	Post20	Pre	Post0	Post20
Fundamental	LF	0.562 ± 0.036	0.582 ± 0.034	0.572 ± 0.035	0.558 ± 0.036	0.570 ± 0.034	0.574 ± 0.035
	MF	0.498 ± 0.043	0.538 ± 0.039	0.506 ± 0.040	0.536 ± 0.039	0.515 ± 0.039	0.496 ± 0.040
	HF	0.300 ± 0.023	0.316 ± 0.023	0.305 ± 0.023	0.312 ± 0.023	0.305 ± 0.023	0.301 ± 0.023
	SHF	0.181 ± 0.014	0.197 ± 0.014	0.190 ± 0.015	0.184 ± 0.014	0.182 ± 0.015	0.178 ± 0.015
Harmonic	LF	0.302 ± 0.019	0.311 ± 0.018	0.315 ± 0.022	0.314 ± 0.022	0.314 ± 0.021	0.335 ± 0.024
	MF	0.201 ± 0.016	0.210 ± 0.015	0.215 ± 0.018	0.210 ± 0.019	0.208 ± 0.018	0.209 ± 0.018
	HF	0.125 ± 0.007	0.133 ± 0.008	0.132 ± 0.008	0.135 ± 0.010	0.136 ± 0.010	0.140 ± 0.010
	SHF	0.076 ± 0.003	0.084 ± 0.003	0.080 ± 0.003	0.080 ± 0.004	0.079 ± 0.003	0.080 ± 0.004

Table S7: LMM results of SNR for the four frequency bands

	**Main effect: Time**	**Main effect: Condition**	**Interaction: Time×Condition**
LF	$F(1,1916) = 0.194, p = 0.660$	$F(1,1916) = 11.779, p = 6.116\text{E}{-4}$	$F(1,1916) = 0.005, p = 0.947$
MF	$F(1,1916) = 8.495, p = 0.004$	$F(1,1916) = 50.699, p = 1.517\text{E}{-12}$	$F(1,1916) = 22.042, p = 2.857\text{E}{-6}$
HF	$F(1,1916) = 0.309, p = 0.578$	$F(1,1916) = 6.667, p = 0.010$	$F(1,1916) = 3.663, p = 0.056$
SHF	$F(1,1916) = 0.528, p = 0.467$	$F(1,1916) = 22.264, p = 2.548\text{E}{-6}$	$F(1,1916) = 3.887, p = 0.049$

Table S8: SNR of SSVEP across four bands (mean ± SE dB)

Band	Real			Sham		
	Pre	Post0	Post20	Pre	Post0	Post20
LF	-12.081 ± 0.558	-12.161 ± 0.553	-12.506 ± 0.557	-12.721 ± 0.564	-12.784 ± 0.557	-12.712 ± 0.556
MF	-10.858 ± 0.545	-10.348 ± 0.512	-11.343 ± 0.537	-10.942 ± 0.447	-11.593 ± 0.553	-11.531 ± 0.469
HF	-10.632 ± 0.510	-10.729 ± 0.565	-11.215 ± 0.571	-10.611 ± 0.500	-11.178 ± 0.630	-11.355 ± 0.614
SHF	-12.959 ± 0.513	-12.825 ± 0.428	-13.690 ± 0.444	-13.316 ± 0.511	-13.696 ± 0.546	-13.347 ± 0.437

Table S9: Spectrum power of task-related and task-unrelated components (mean ± SE μV^2)

Component	Band	Real			Sham		
		Pre	Post0	Post20	Pre	Post0	Post20
Task-related	LF	1.403 ± 0.099	1.386 ± 0.088	1.433 ± 0.119	1.203 ± 0.068	1.243 ± 0.080	1.363 ± 0.085
	MF	1.091 ± 0.072	1.058 ± 0.063	0.961 ± 0.065	1.118 ± 0.076	0.968 ± 0.064	0.979 ± 0.065
	HF	0.427 ± 0.026	0.394 ± 0.022	0.401 ± 0.024	0.410 ± 0.020	0.395 ± 0.021	0.385 ± 0.019
	SHF	0.096 ± 0.005	0.098 ± 0.005	0.078 ± 0.004	0.092 ± 0.005	0.093 ± 0.005	0.089 ± 0.005
Task-unrelated	LF	2.248 ± 0.104	2.653 ± 0.162	2.599 ± 0.176	2.491 ± 0.163	2.535 ± 0.128	2.875 ± 0.185
	MF	1.215 ± 0.051	1.147 ± 0.046	1.163 ± 0.050	1.316 ± 0.061	1.225 ± 0.058	1.233 ± 0.056
	HF	0.537 ± 0.023	0.499 ± 0.021	0.524 ± 0.024	0.534 ± 0.021	0.514 ± 0.020	0.533 ± 0.022
	SHF	0.190 ± 0.006	0.189 ± 0.006	0.182 ± 0.006	0.186 ± 0.007	0.190 ± 0.008	0.189 ± 0.008

Table S10: Two-way rm-ANOVA results for microstate analysis during the SSVEP task

	**Main effect: Time**	**Main effect: Microstate**	**Interaction: Time×Microstate**
Real-Duration	$F(1,23) = 0.726, p = 0.403$	$F(3,69) = 2.117, p = 0.106$	$F(3,69) = 5.666, p = 0.002$
Real-Occurrence	$F(1,23) = 0.024, p = 0.878$	$F(3,69) = 1.575, p = 0.203$	$F(3,69) = 13.453, p = 5.244\text{E}{-7}$
Real-Contribution	$F(1,23) = 0.590, p = 0.450$	$F(3,69) = 1.461, p = 0.233$	$F(3,69) = 9.484, p = 2.511\text{E}{-5}$
Sham-Duration	$F(1,23) = 0.322, p = 0.576$	$F(3,69) = 2.093, p = 0.109$	$F(3,69) = 1.681, p = 0.179$
Sham-Occurrence	$F(1,23) = 0.210, p = 0.651$	$F(3,69) = 0.494, p = 0.687$	$F(3,69) = 1.342, p = 0.268$
Sham-Contribution	$F(1,23) = 0.432, p = 0.518$	$F(3,69) = 0.913, p = 0.440$	$F(3,69) = 1.065, p = 0.370$

Table S11: Two-way rm-ANOVA results for microstate analysis during the resting state

	**Main effect: Time**	**Main effect: Microstate**	**Interaction: Time×Microstate**
Real-Duration	$F(1,23) = 0.957, p = 0.338$	$F(3,69) = 0.649, p = 0.586$	$F(3,69) = 1.548, p = 0.210$
Real-Occurrence	$F(1,23) = 1.445, p = 0.242$	$F(3,69) = 0.580, p = 0.630$	$F(3,69) = 6.273, p = 7.923\text{E}{-4}$
Real-Contribution	$F(1,23) = 0.519, p = 0.479$	$F(3,69) = 0.031, p = 0.992$	$F(3,69) = 4.235, p = 0.008$
Sham-Duration	$F(1,23) = 1.334, p = 0.260$	$F(3,69) = 0.767, p = 0.516$	$F(3,69) = 1.570, p = 0.204$
Sham-Occurrence	$F(1,23) = 2.320, p = 0.141$	$F(3,69) = 3.581, p = 0.018$	$F(3,69) = 1.303, p = 0.281$
Sham-Contribution	$F(1,23) = 4.726\text{E}{-18}, p = 1.000$	$F(3,69) = 1.165, p = 0.329$	$F(3,69) = 1.680, p = 0.179$

Table S12: Rm-ANOVA results for Compact-CNN method in four frequency bands

	**Main effect: Time**	**Main effect: Condition**	**Interaction: Time×Condition**
LF	$F(1,23) = 2.120, p = 0.159$	$F(1,23) = 0.088, p = 0.770$	$F(1,23) = 0.003, p = 0.960$
MF	$F(1,23) = 3.150, p = 0.089$	$F(1,23) = 2.316, p = 0.142$	$F(1,23) = 8.161, p = 0.009$
HF	$F(1,23) = 0.326, p = 0.574$	$F(1,23) = 0.850, p = 0.366$	$F(1,23) = 2.218, p = 0.150$
SHF	$F(1,23) = 0.705, p = 0.410$	$F(1,23) = 2.355, p = 0.139$	$F(1,23) = 0.730, p = 0.402$

Table S13: Rm-ANOVA results for Conv-CA method in four frequency bands

	**Main effect: Time**	**Main effect: Condition**	**Interaction: Time×Condition**
LF	$F(1,23) = 0.143, p = 0.709$	$F(1,23) = 0.154, p = 0.698$	$F(1,23) = 0.864, p = 0.362$
MF	$F(1,23) = 2.399, p = 0.135$	$F(1,23) = 2.029, p = 0.168$	$F(1,23) = 4.641, p = 0.042$
HF	$F(1,23) = 0.113, p = 0.740$	$F(1,23) = 4.193, p = 0.052$	$F(1,23) = 4.680, p = 0.041$
SHF	$F(1,23) = 4.628, p = 0.042$	$F(1,23) = 0.964, p = 0.336$	$F(1,23) = 0.080, p = 0.780$

Table S14: Rm-ANOVA results for ConsenNet method in four frequency bands

	**Main effect: Time**	**Main effect: Condition**	**Interaction: Time×Condition**
LF	$F(1,23) = 1.094, p = 0.306$	$F(1,23) = 3.419, p = 0.077$	$F(1,23) = 0.070, p = 0.794$
MF	$F(1,23) = 0.238, p = 0.630$	$F(1,23) = 3.933, p = 0.059$	$F(1,23) = 4.612, p = 0.043$
HF	$F(1,23) = 0.583, p = 0.453$	$F(1,23) = 0.314, p = 0.580$	$F(1,23) = 4.594, p = 0.043$
SHF	$F(1,23) = 1.682, p = 0.208$	$F(1,23) = 0.127, p = 0.725$	$F(1,23) = 0.617, p = 0.440$

Table S15: Decoding accuracy with three deep learning methods across four frequency bands under 0.5s data length (mean ± SE %)

		**Real**			**Sham**		
Method	Band	Pre	Post0	Post20	Pre	Post0	Post20
Compact-CNN	LF	47.500 ± 3.986	43.333 ± 3.381	42.708 ± 4.228	48.125 ± 4.308	44.167 ± 3.871	42.708 ± 4.256
	MF	57.292 ± 4.166	66.667 ± 4.225	60.417 ± 4.448	56.250 ± 53088	56.458 ± 4.728	57.708 ± 4.965
	HF	50.625 ± 4.817	55.625 ± 4.637	49.167 ± 4.962	51.667 ± 4.236	49.167 ± 4.446	47.500 ± 4.135
	SHF	35.417 ± 2.930	35.000 ± 3.785	29.583 ± 2.392	31.667 ± 3.688	28.542 ± 3.429	30.833 ± 2.743
Conv-CA	LF	76.042 ± 4.269	79.792 ± 4.109	68.750 ± 5.314	77.500 ± 4.156	75.625 ± 4.303	71.875 ± 4.804
	MF	73.542 ± 4.098	83.542 ± 2.291	76.042 ± 3.864	74.583 ± 3.213	71.667 ± 4.655	74.167 ± 4.775
	HF	73.750 ± 5.206	81.042 ± 3.964	75.208 ± 4.404	73.125 ± 4.476	68.750 ± 4.843	64.375 ± 6.040
	SHF	50.625 ± 4.481	54.167 ± 3.916	54.083 ± 4.050	46.250 ± 4.985	51.042 ± 4.677	67.875 ± 4.610
ConsenNet	LF	84.167 ± 3.085	86.875 ± 2.838	80.208 ± 3.541	80.208 ± 3.914	82.083 ± 3.941	80.208 ± 3.836
	MF	84.792 ± 3.234	90.625 ± 2.069	84.583 ± 3.103	83.958 ± 2.568	79.583 ± 3.907	81.250 ± 4.169
	HF	77.917 ± 5.464	85.000 ± 2.946	81.042 ± 3.807	81.250 ± 4.021	77.708 ± 4.310	74.375 ± 4.763
	SHF	56.875 ± 4.849	62.292 ± 5.052	54.167 ± 5.422	57.292 ± 4.778	58.542 ± 4.755	54.375 ± 5.106

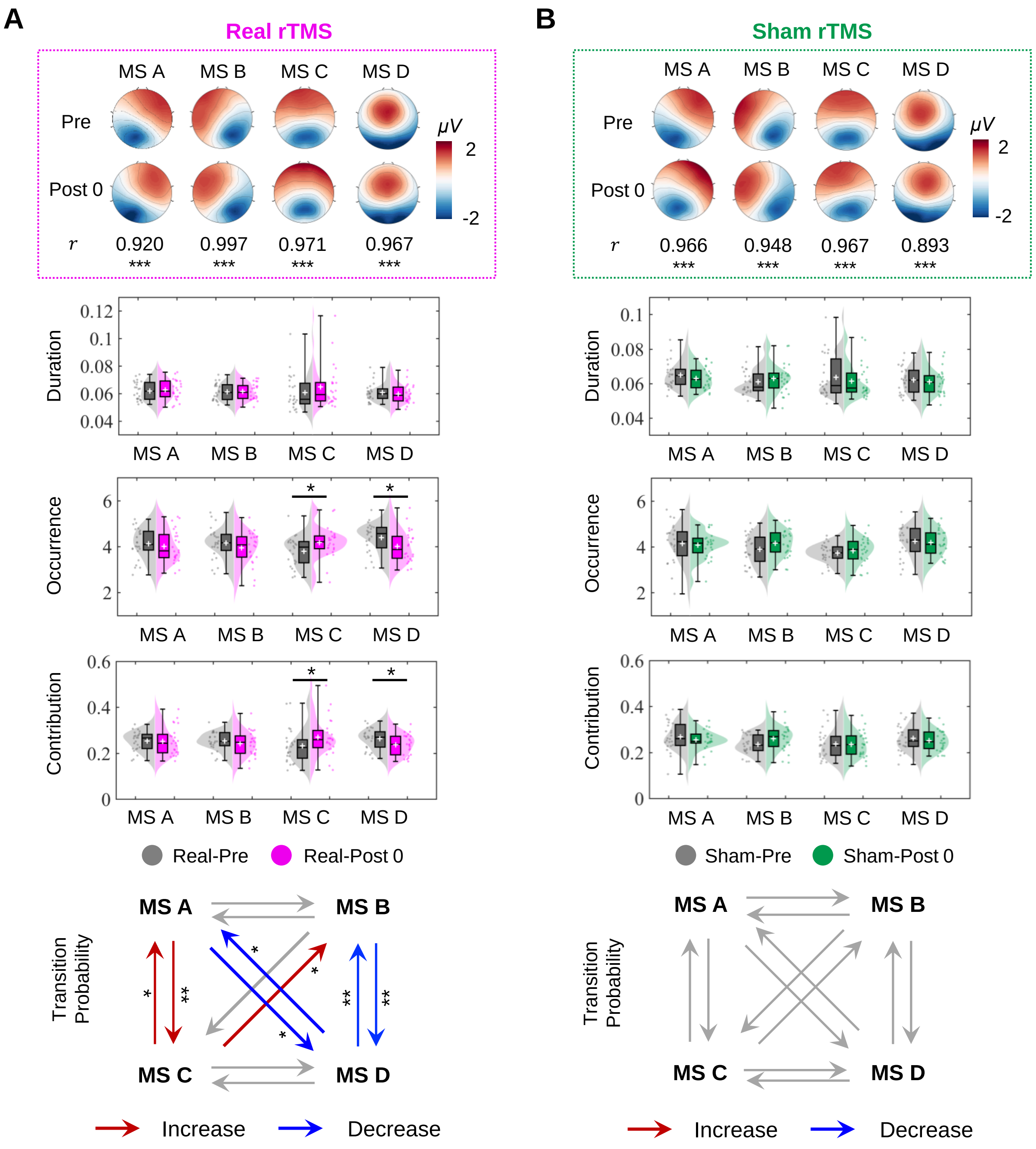

Figure S1: Microstate analyses results for resting EEG at Pre and Post0 under real and sham conditions. (A) Microstate configurations under the real condition. (B) Microstate configuration under the sham condition. Four microstate classes (A-D) explained 71.20%, 74.18%, 73.57%, and 74.88% of the global variance for the Real-Pre and Real-Post0, Sham-Pre, and Sham-Post0, respectively. A two-way rm-ANOVA revealed significant Time×Microstate Class interactions for occurrence and contribution under the real condition. One-tailed paired t-tests ($n = 24$) indicated significant increases in occurrence and contribution for microstate C, alongside significant decreases in occurrence and contribution for microstate D. Real rTMS significantly increased transitions between A and C (A $\rightarrow$ C: $p < 0.01$, C $\rightarrow$ A: $p < 0.05$) and from C to B ($p < 0.05$), while it decreased transitions between B and D (both: $p < 0.01$) and between A and D (both: $p < 0.05$).
